# Leukocytes infiltration correlates intratumoral microvessel density and influence overall and late-phase disease-free survival in hepatocellular carcinoma

**DOI:** 10.1097/MD.0000000000028135

**Published:** 2021-12-03

**Authors:** Yuan Yang, Ning Fu, Haiqing Wang, Jingcheng Hao

**Affiliations:** a Department of Rheumatology and Immunology, The First Affiliated Hospital of Chengdu Medical College, Chengdu, Sichuan, PR China; b Department of Hepatobiliary and Vascular Surgery, The First Affiliated Hospital of Chengdu Medical College, Chengdu, Sichuan, PR China; c Department of Hepatobiliary and Pancreatic Surgery, Sichuan Cancer Hospital & Institute, Chengdu, Sichuan, PR China.

**Keywords:** hepatectomy, hepatocellular carcinoma, intratumoral microvessel density, prognosis, tumor-infiltrating leukocytes

## Abstract

Hepatocellular carcinoma (HCC) is a severe type of primary liver cancer with high postoperative recurrence. The prognosis predictability of tumor-infiltrating leukocytes (TILs) for patients who underwent HCC resection has been widely reported. However, limited information is available about TIL trafficking, which is also crucial for HCC patients.

We included tumor tissue samples and clinical data from 89 HCC patients in this study and performed immunohistochemistry for CD3, CD8, FoxP3, and CD31. TILs were measured using an algorithm for quantification of tumor immune stroma (QTiS). Intratumoral microvessels were counted using Weidner's method. We first examined correlations among them and analyzed their relationships with clinical and survival data.

Intratumoral microvessel density (iMVD) was significantly correlated with infiltration of CD3+ (*r* = 0.338, *P* = .001) and CD8+ (*r* = 0.320, *P* = .002) cells, but not FoxP3+ (*r* = 0.153, *P* = .152) cells. After multivariate analysis, higher infiltration of CD3+ (*P* = .038) independently showed significant predictability on better overall survival after resection of HCC. Although no influence of CD3+ (*P* = .386) and CD8+ (*P* = .648) cells were found on general disease-free survival, infiltration of CD3+ (*P* = .012), tumor size (*P* = .032) and albumin (*P* = .007) cells independently predicted late-phase disease-free survival. No significant relationships regarding iMVD, and infiltration of FoxP3+ cells with overall and disease-free survival were found.

Our data suggest that increased iMVD could enrich tumor-infiltrating CD3+ cells. Infiltrated CD3+ cells could help to better predict both the overall and late-phase disease-free survival after resection of HCC.

## 1 Introduction

Hepatocellular carcinoma (HCC) is a dominant type of liver malignancy. It is also the second principal cause of cancer-related fatality worldwide.^[1]^ Hepatectomy is the most efficient curative treatment for HCC. Despite the unceasing improvement of resection techniques, postoperative recurrence rate can reach up to 70% in 5 years, and this recurrence leads to a high rate of death in HCC patients.^[2]^ Therefore, it is essential to improve strategies to predict and prevent postoperative HCC recurrence.

Immunology has been shown to play a significant role in malignancy development.^[3]^ Changes in the immune system have been investigated in HCC for many years.^[3]^ In previous studies, tumor-infiltrating leukocytes (TILs), especially those infiltrating CD3+ and CD8+ cells, have been extensively reported as predictors for survival in numerous human solid malignant tumors.^[4,5]^ Moreover, the predictabilities of these infiltrating leukocytes are suggested to be better than that of conventional clinical staging systems.^[6]^

Not only does the type and amount of TILs, but also the trafficking needs to be deliberated. Neovascularized arteries mainly supply to the HCCs.^[7]^ Neovascularization is necessary for cancer development by delivering nutrition and oxygen. However, it could also possibly enhance leukocyte infiltration, which in theory is reliant on intratumoral blood vessels. Furthermore, the strong correlations between endothelial venules and leukocyte infiltration have been proven in many human solid malignancies, such as melanomas, breast, ovarian, colon, and lung carcinomas.^[8]^ In particular, these high endothelial venules conferred better survival in invasive breast cancer. Additionally, animal studies have shown that regulatory T cells (Tregs), which could promote tumor progression by their immunosuppressive abilities, could also stimulate neovasulucariztion.^[9]^ However, few studies have provided similar information regarding HCC.

In the present study, we investigated the expression of total T cells, cytotoxic T cells, regulatory T cells, and intratumoral microvessel density (iMVD) in resected HCC tissues, the potential correlations among them, and their relationships to clinical data.

## 2 Material & methods

### 2.1 Patients and clinical data

We collected tumor tissues samples from 89 curatively resected HCC patients from October 2013 to November 2017. The diagnoses of all patients were in accordance with the guidelines for diagnosis and treatment of primary liver cancer in China.^[10,11]^ Clinical diagnoses of all cases were confirmed histologically after resection. All tumor samples were anonymously coded during the experimental procedure. This study was approved by the institutional review board of Chengdu Medical College, China. Data collection and experiments were carried out in accordance with the ethical guidelines of the Declaration of Helsinki. Informed consent for data and tissue collection from all study subjects (if subjects were under 18 years of age, from corresponding legal guardians) was obtained at admission.

We collected the following epidemiological and clinical data from medical records in our database for statistics: gender, age at operation, history of hepatitis, presence of cirrhosis, tumor size and number in preoperative imaging, microvascular and macrovascular invasion, Barcelona clinic liver cancer (BCLC) staging,^[12]^ Milan staging^[13]^, routine laboratory examination results including white blood cell count, hemoglobin, platelet count, alanine transaminase (ALT), aspartate transaminase, bilirubin, albumin, and serum alpha-fetoprotein (AFP) levels.

### 2.2 Immunohistochemical staining

We performed immunohistochemical staining as described previously.^[14]^ Four-micrometer sections of paraffin blocks were prepared for staining. Dewaxing and hydration were performed with xylene, ethanol, and distilled water. Antigens were retrieved using citrate buffer (pH 6.0) or EDTA solution (pH 8.0). Following incubation with antibodies for CD3 (ab5690, Abcam Plc.), CD8 (ab4055, Abcam Plc.), FoxP3 (ab4055, Abcam Plc.), and CD31 (ab28364, Abcam Plc.), incubation with secondary antibodies was carried out. 3, 3-Diaminobenzidine method was used for staining. Counterpart staining was performed using Hemalaun. Negative and isotype controls were used as appropriate.

### 2.3 Analysis of immunohistochemical staining

#### 2.3.1 Quantification of tumor-infiltrating leukocytes

TILs were quantified using an algorithm for quantification of tumor immune stroma (QTiS).^[15]^ Hotspots were defined as areas with the highest infiltration density and were chosen under a low-power magnification (40 × ). In accordance with the QTiS algorithm, we analyzed the mean amount of the three hotspots. The images were captured under high-power magnification (Nikon Eclipse Ci with Nikon Plan, 200 × ). The number of positive cells per image was counted using ImageJ software (version 1.52p, National Institutes of Health; Bethesda, MD, USA) with threshold adjustment for further analysis.

#### 2.3.2 Quantification of intratumoral vessels

To measure intratumoral vessels, we adopted the method published by Weidner in 1995.^[16]^ This method is also a quantificational strategy that is based on “hotspot-choosing,” which is similar to the QTiS we used for TILs. We scanned the slides under a low-power magnification (40 × ) and selected those regions with the highest number of intratumoral vessels as hotspots. We also analyzed the mean amount of 3 hotspots that were captured under high-power magnification (200 × ). In accordance with Weidner's method, we counted the number of vessels instead of stained cells.

### 2.4 Statistical analysis

The distribution of continuous data was inspected by Shapiro–Wilk test. Continuous numbers were presented as mean ± SD (normal distribution) or median (interquartile range [IQR]) (skewed distribution), and compared using an independent *t*-test (normal distribution) or Mann–Whitney *U* test (normal distribution). We compared contingency data using χ^2^-test or Fisher exact test, and assessed the correlation by calculating Pearson's *r* values. For survival analysis of continuous data, we grouped the patients into high (>medians) or low (≤medians) groups using the median of each variable, followed by a log-rank test. Variables with a level of significance less than or equal to 0.100 were then included in a multivariate analysis by stepwise Cox regression. Disease-free survival was defined as the time after surgery without any tumor recurrence. Patients were further divided into an early-phase recurrence group with recurrence recorded within 2 years of surgery, and a late-phase group without any recurrence recorded within 2 years of surgery. We defined overall survival as the time from operation till death. Univariate analysis was conducted to screen for potential variables. All statistical calculations were performed using Prism (version 8.2.0, GraphPad, La Jolla, CA) and SPSS software (version 25.0.0.2, IBM Corp, Armonk, NY). We considered statistical significance at *P* < .050.

## 3 Results

### 3.1 Analysis of demographics of the study population

**Figure 1:**
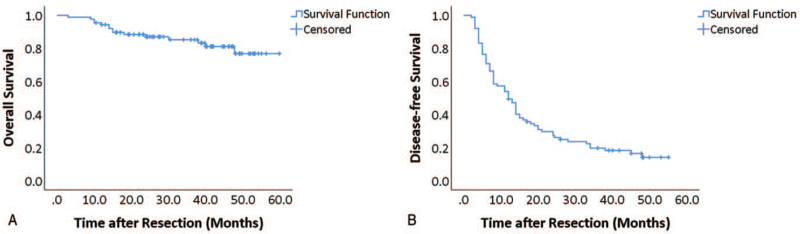
Overall (A) and disease-free (B) survival of all patients. Kaplan–Meier curves for overall (time from resection to death) and disease-free (time from resection to recurrence) survival of all patients.

**Figure 2:**
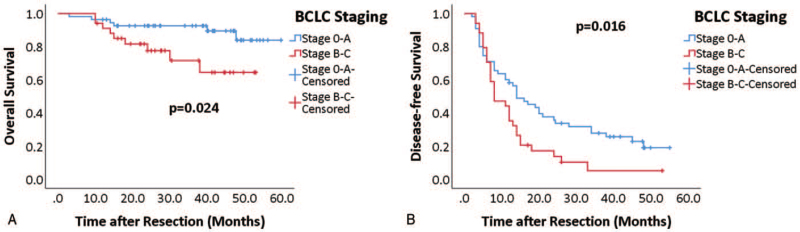
BCLC (Barcelona clinic liver cancer) stage on overall (A) and disease-free (B) survival of all patients. Kaplan–Meier curves for overall (time from resection to death) and disease-free (time from resection to recurrence) survival for BCLC stages 0–A (blue curve) and B–C (red curve). Log-rank test was used to compare significance.

Eighty nine resected HCC tissue samples were included in the current study. Of the patients, 86.5% were male. The median age of the sampled patients was 52 (18.50) years. Patients had a history of hepatitis B infection (79.8%) and liver cirrhosis (70.8%), which is a typical representative of the eastern population. Twelve patients (13.5%) had multiple tumor lesions as seen in preoperative imaging. Five (5.6%) and 6 (6.7%) patients had microvascular and macrovascular invasions, respectively, as observed in postoperative pathological examination. Of the 89 patients, 50 (56.2%) patients were classified as BCLC stage A, 5 (5.6%), 15 (16.9%), and 19 (21.3%) patients were classified as stage 0, B, and C, respectively. Fifty three patients (59.6%) were beyond the Milan criteria. We have summarized the demographic data in Table 1. The median follow-up after resection was 36.97 months. The estimated cumulative proportion of overall survival at 1, 3, and 5 years was 89.7%, 87.0%, and 81.1%, respectively, and that of disease-free survival was 92.1%, 66.3%, and 34.5%, respectively (Fig. 1). During the follow-up period, 73 (82.0%) patients showed postoperative recurrence, and these recurrent patients had a significantly higher proportion of hepatitis infection (63, 86.3%) in comparison with those patients without recurrence (8, 50.0%, *P* = .003). We did not observe any other significant demographical differences regarding the recurrence. (Table 1) Fifty four (74.0%) of all recurrent patients had undergone certain treatments; these were, radiofrequency ablation (29 cases, 39.7%), repeat hepatectomy (17 cases, 23.3%), and transarterial chemoembolization (8 cases, 11.0%). Following the BCLC staging, patients were divided into 2 groups (stage 0-A, and stage B-C) for Kaplan–Meier survival analysis. We found that patients with BCLC stage 0-A had a significantly better overall survival (Fig. 2A, *P* = .024) and disease-free survival (Fig. 2B, *P* = .016) than those with stage B-C. Therefore, we considered our cohort to be representative of eastern HCC population with acceptable sample size for further investigation.

**Table 1:** Demographics of the study cohort.

Variables	All (n = 89)	With recurrence(n = 73)	Without recurrence(n = 16)	*P* value
Gender(Male/Female)	77 (86.5%)/12 (13.5%)	63 (86.3%)/10 (13.7%)	14 (87.5%)/2 (12.5%)	1.000
Age (Years)(Median (IQR))	52.00 (18.50)	52.00 (18.00)	53.00 (24.00)	.600
HBV	71 (79.8%)	63 (86.3%)	8 (50.0%)	.003
Cirrhosis	63 (70.8%)	49 (67.1%)	14 (87.5%)	.135
AFP (ng/mL)(Media00n (IQR))	67.00 (874.50)	80.00 (874.50)	21.36 (767.50)	.304
Tumor Size (cm)(Median (IQR))	5.00 (5.00)	5.00 (5.00)	4.50 (3.75)	.348
Tumor Multiplicity	12 (13.5%)	11 (15.1%)	1 (6.3%)	.686
Microvascular Invasion	5 (5.6%)	5 (6.8%)	0 (0.0%)	.580
Macrovascular Invasion	6 (6.7%)	6 (8.2%)	0 (0.0%)	.586
BCLC Stage(0/A/B/C)	5 (5.6%)/50 (56.2%)/15 (16.9%)/19 (21.3%)	5 (6.8%)/37 (50.7%)/13 (17.8%)/18 (24.7%)	0 (0.0%)/13 (81.3%)/2 (12.5%)/1 (6.3%)	.134
Beyond Milan Criteria	53 (59.6%)	45 (61.6%)	8 (50.0%)	.412
Albumin (g/L)(Median (IQR))	41.00 (5.85)	41.00 (5.45)	43.00 (6.75)	.292
ALT (U/L)(Median (IQR))	37.00 (39.00)	38.00 (42.00)	32.50 (19.25)	.435
AST (U/L)(Median (IQR))	37.00 (30.50)	38.00 (31.00)	32.50 (12.00)	.318
Bilirubin (μmol/L)(Median (IQR))	17.00 (8.50)	17.10 (8.35)	16.55 (8.80)	.575
WBC (10–9/L)(Median (IQR))	5.37 (2.75)	5.33 (2.26)	5.97 (3.90)	0.525
HGB (g/L)(Median (IQR))	139.00 (23.50)	140.00 (27.00)	133.00 (16.75)	.129
PLT (10–9/L)(Median (IQR))	124.00 (86.00)	124.00 (90.00)	108.50 (87.25)	.642
CD3 (cells/HPF)(Median (IQR))	86.00 (145.83)	70.67 (123.83)	166.50 (179.25)	.012
CD8 (cells/HPF)(Median (IQR))	35.00 (64.67)	31.00 (60.67)	66.00 (88.92)	.062
FoxP3 (cells/HPF)(Median (IQR))	11.00 (23.67)	9.00 (22.33)	17.50 (26.58)	.323
iMVD (vessels/HPF)(Median (IQR))	26.67 (22.83)	26.00 (24.17)	32.33 (19.58)	.240

### 3.2 Intratumoral microvessel density correlates with tumor-infiltrating leukocytes

**Figure 3:**
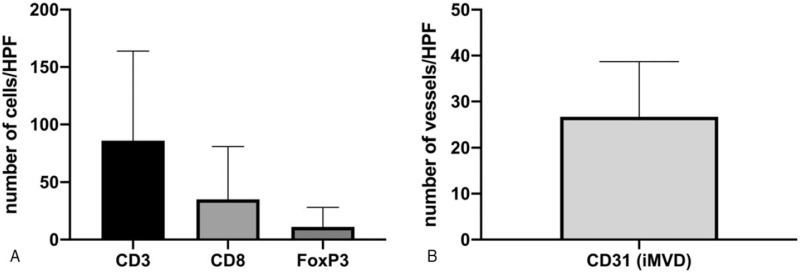
The expression analysis of CD3, CD8, FoxP3 (A), and CD31 (B) of all patients. For each case, average number of cells or vessels were calculated from three representative HPFs (high power field). Data for all patients were presented as median (interquartile range).

**Figure 4:**
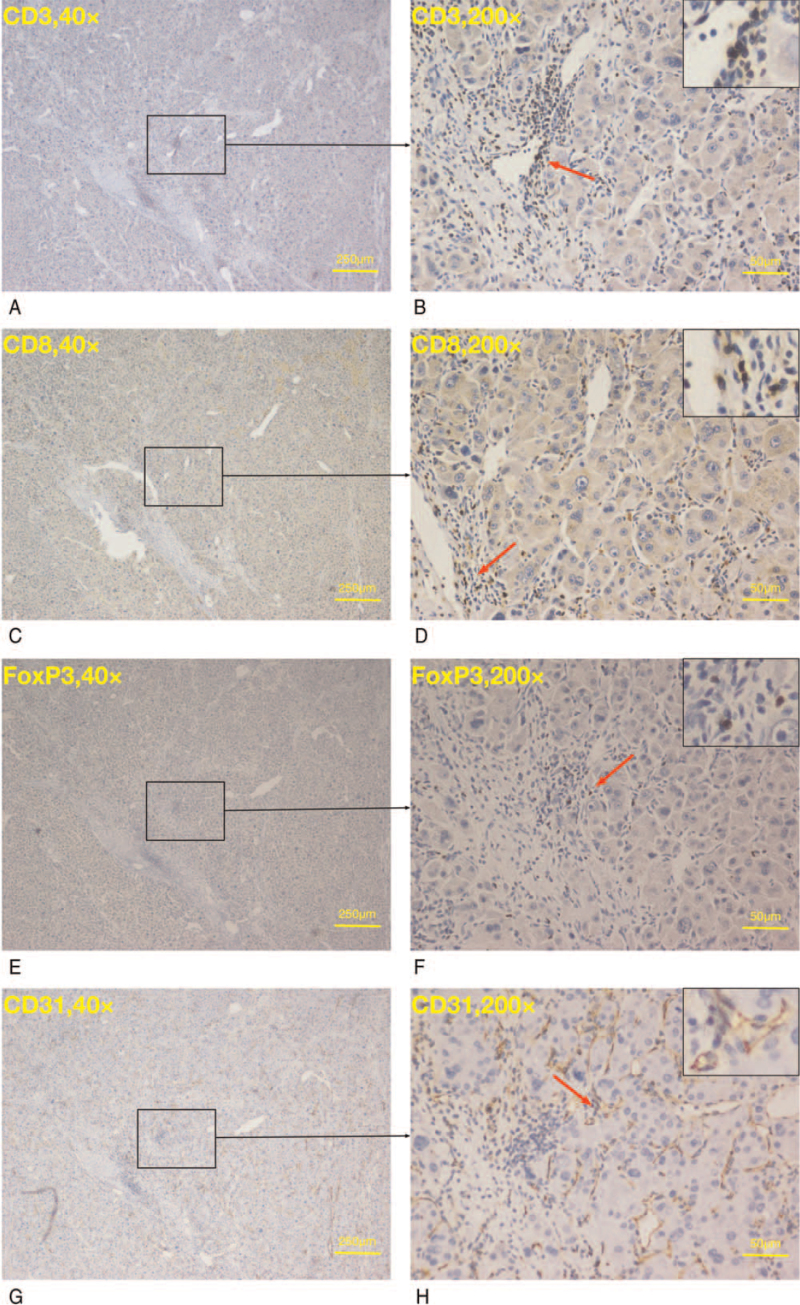
Representative expression of CD3, CD8, FoxP3, and CD31 cells under 40 × , and 200 × magnifications.

Quantification of TILs showed that in our current cohort, the median (IQR) cell density for CD3+, CD8+, and FoxP3+ were 86.00 (145.83) cells/high power field (HPF) (High Power Field, 200 × ), 35.00 (64.67) cells/HPF, and 11.00 (23.67) cells/HPF, respectively (Fig. 3A). The median (IQR) iMVD was 26.67 (22.83) vessels/HPF (Fig. 3B). We observed a significantly higher number of CD3+ cells in patients without recurrence (166.50 [179.25]) compared with those with recurrence (70.67 [123.83], *P* = .012), as well as a higher tendency for CD8+ cells (66.00 [88.92] vs 31.00 (60.67), *P* = .062). No significant difference was observed in FoxP3+ cells (*P* = .323) and iMVD (P = .240) (Table 1). Figure 4 shows representative staining of CD3, CD8, FoxP3, and CD31 on similar location from 1 patient.

**Figure 5:**
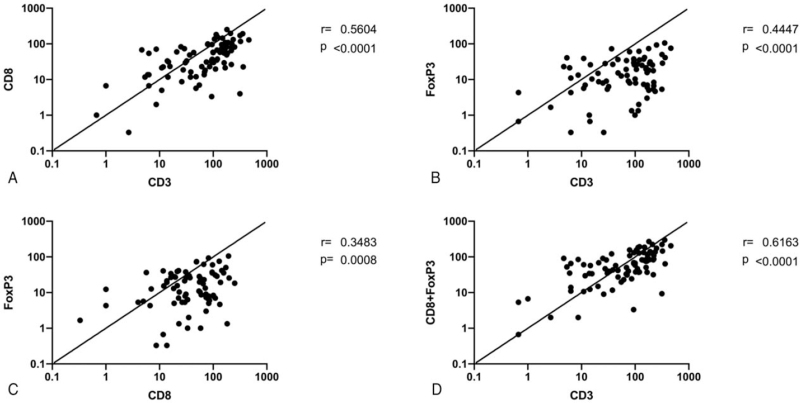
Correlations among CD3, CD8, and FoxP3. Correlations were assessed by calculating Pearson's *r* values.

Further, correlation analysis showed that the infiltration of CD3+ cells significantly correlated with CD8+ (*r* = 0.560, *P* < .001) (Fig. 5A) and FoxP3+ cells (*r* = 0.445, *P* < .001) (Fig. 5B). In addition, infiltration of CD8+ cells was significantly correlated with FoxP3+ cells (*r* = 0.348, *P* = .001) (Fig. 5C). Due to the fact that CD8+ and FoxP3+ cells (commonly known as Cytotoxic T cells and Regulatory T cells) are 2 major subtypes of CD3+ cells (commonly known as Total T cells), we further obtained a stronger correlation value between the infiltration of CD3+ cells and the summary of CD8+ and FoxP3+ cells (*r* = 0.616, *P* < .001) (Fig. 5D).

**Figure 6:**
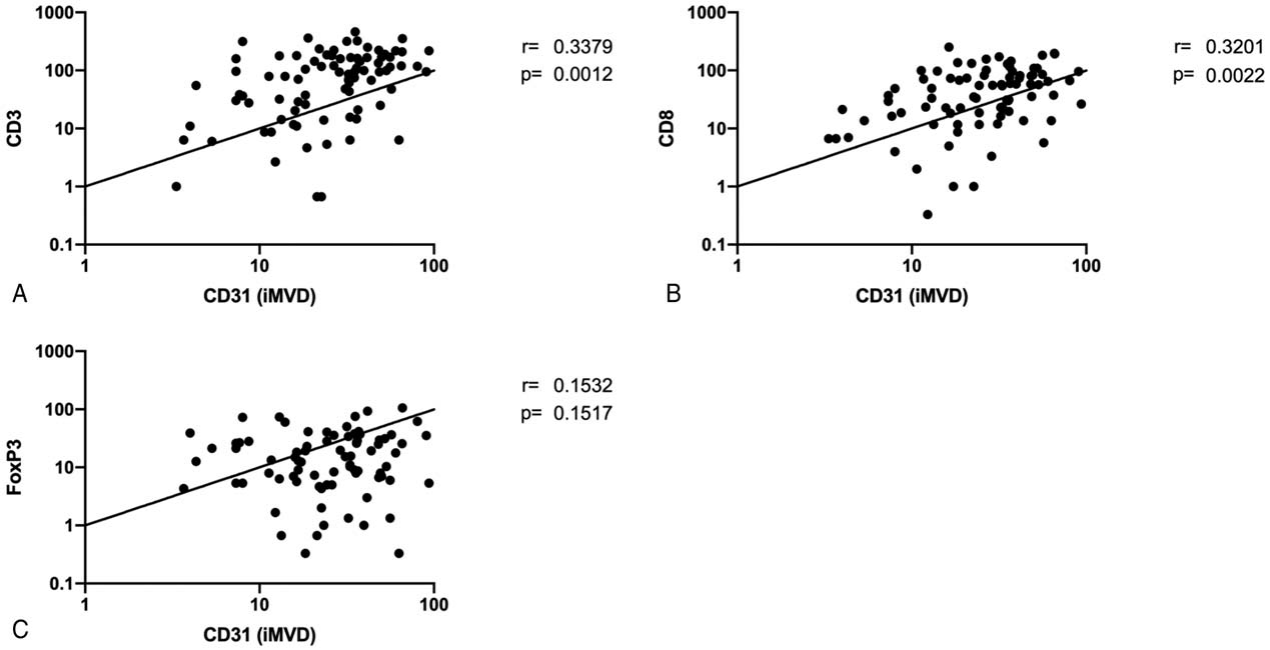
Correlation between iMVD and CD3 (A), CD8 (B), and FoxP3 (C). Correlations were assessed by calculating Pearson's *r* values.

Correlation analysis between iMVD and TILs revealed significant correlations between iMVD and CD3+ cells (*r* = 0.338, *P* = .001) (Fig. 6A) as well as between iMVD and CD8+ cells (*r* = 0.320, *P* = .002) (Fig. 6B). However, no significant correlation was found between iMVD and FoxP3+ cells (*r* = 0.153, *P* = .152) (Fig. 6C).

### 3.3 Infiltration of CD3+ predicts overall survival

In the analysis of demographic and routine laboratory data, we found that high infiltration of CD3+ cells was significantly related to small tumor size (*P* = .026), low macrovascular invasion (*P* = .026), low BCLC stage (*P* = .009), and low platelet count (*P* = .040). High infiltration of FoxP3+ cells was significantly related to high serum AFP (*P* = .005) and high iMVD was significantly related to less macrovascular invasion (*P* = .012). Infiltration of CD8+ cells was not significantly related to any variables in the data. (Supplemental Digital Content Table S1)

**Figure 7:**
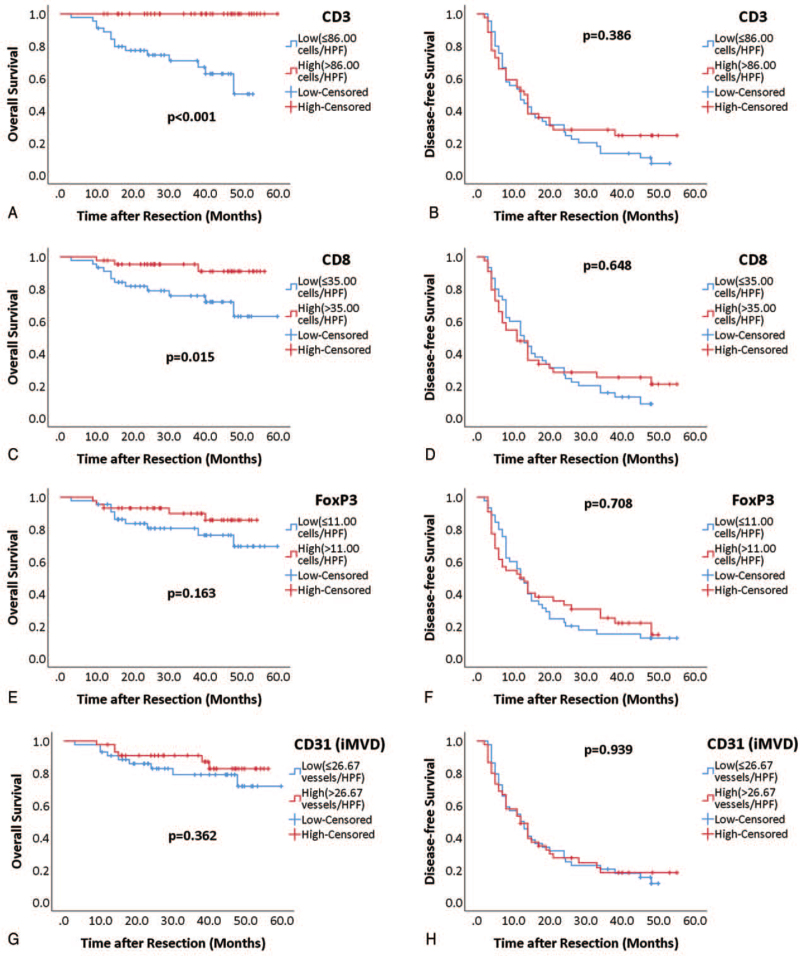
Kaplan–Meier curves of infiltration of CD3+ (A, B), CD8+ (C, D), FoxP3+ (E, F) cells, and iMVD (G, H) for overall and disease-free survival. For Kaplan–Meier analysis, patients were grouped as high (red curve) or low (blue curve), according to medians. Log-rank test was used to compare significance.

From the results of Kaplan–Meier analysis, high infiltration of CD3+ (*P* < .001) (Fig. 7A) and CD8+ cells (*P* = .015) (Fig. 7C) predicted better overall survival. Infiltration of FoxP3+ cells (*P* = .163) (Fig. 7E) and iMVD (*P* = .362) (Fig. 7G) had no significant relationship with overall survival. No significant influence on disease-free survival was found regarding infiltration of CD3+ (*P* = .386) (Fig. 7B), CD8+ (*P* = .648) (Fig.7D), FoxP3+ cells (*P* = .708) (Fig. 7F), and iMVD (*P* = .939) (Fig. 7H).

In addition, we performed univariate Kaplan–Meier analysis for all demographic variables, and found that BCLC stage (*P* = .024), albumin (*P* = .048), and tendencies for bilirubin (*P* = .062) significantly predicted overall survival. (Table 2) These variables along with CD3+ and CD8+ cells were then included for multivariate Cox regression analysis. After the multivariate statistical analysis, only CD3+ cells remained an independent predictor for overall survival. (Table 3)

**Table 2:** Univariate Kaplan–Meier analysis on overall and disease-free survival.

Variables	Overall survival (*P* values)	Disease-free survival (*P* values)
Gender	.512	.804
Age	.240	.301
HBV	.922	.013
Cirrhosis	.826	.270
AFP	.472	.045
Tumor size	.144	.503
Tumor multiplicity	.552	.262
Microvascular invasion	.103	.024
Macrovascular invasion	.147	.029
BCLC stage	.024	.016
Beyond milan criteria	.190	.305
Albumin	.048	.239
ALT	.420	.026
AST	.135	.409
Bilirubin	.062	.689
WBC	.380	.971
HGB	.443	.309
PLT	.248	.467
CD3+ cells	<.001	.386
CD8+ cells	.015	.648
FoxP3+ cells	.163	.708
iMVD	.362	.939

**Table 3:** Multivariate Cox regression of included variables on overall survival.

Variable	Hazard ratio	95% Confidence interval	*P* value
CD3	0.012	0.001–0.789	.038

Furthermore, hepatitis B (*P* = .013), AFP (*P* = .045), microvascular (*P* = .024) and macrovascular (*P* = .029) invasions, BCLC stage (*P* = .016), and ALT (*P* = .026) were significant predictors for disease-free survival in univariate analyses. (Table 2) Cox regression analysis showed that hepatitis B (*P* = .023), AFP (*P* = .025), microvascular invasion (*P* = .010), and ALT (*P* = .009) were independent predictive values for disease-free survival.(Table 4)

**Table 4:** Multivariate Cox regression of included variables on disease-free survival.

Variables	Hazard ratio	95% Confidence interval	*P* values
HBV	2.200	1.113–4.349	.023
AFP	1.740	1.074–2.818	.025
Microvascular Invasion	3.509	1.359–9.091	.010
ALT	1.934	1.183–3.161	.009

### 3.4 Infiltration of CD3+ predicts late-phase disease-free survival

**Figure 8:**
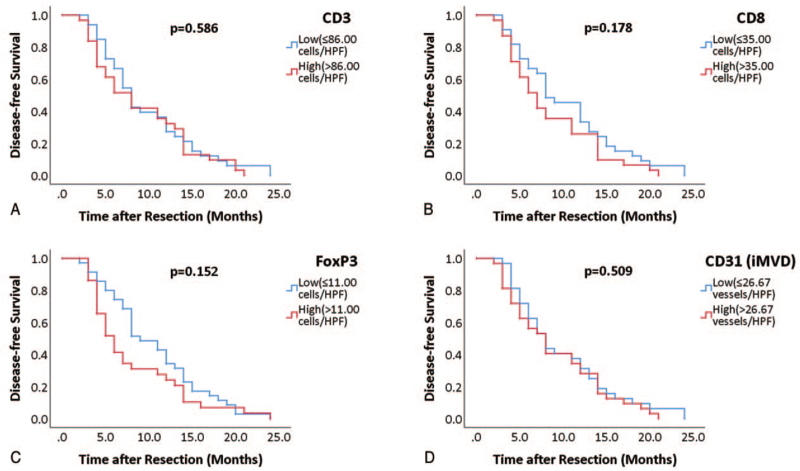
Kaplan–Meier curves of infiltration of CD3+ (A), CD8+ (B), FoxP3+ (C), and iMVD (D) for early-phase disease-free survival. For Kaplan-Meier analysis, patients were grouped as high (red curve) or low (blue curve), according to medians. Log-rank test was used to compare significance.

**Figure 9:**
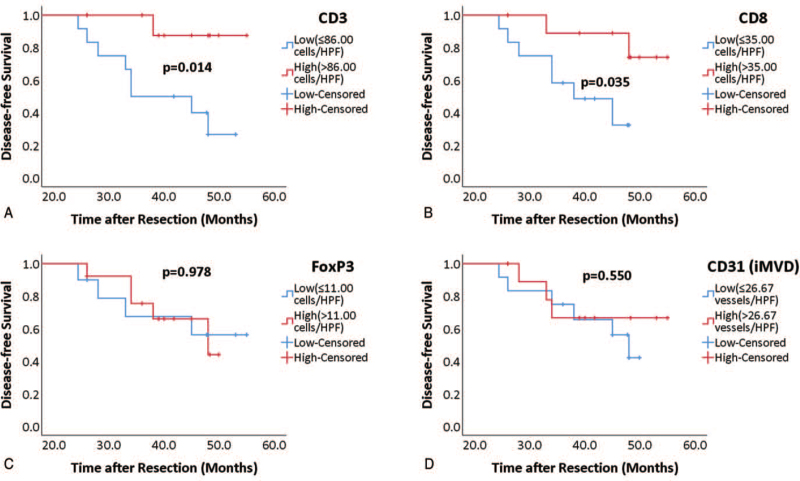
Kaplan–Meier curves of infiltration of CD3+ (A), CD8+ (B), FoxP3+ (C), and iMVD (D) for late-phase disease-free survival. For Kaplan-Meier analysis, patients were grouped as high (red curve) or low (blue curve), according to medians. Log-rank test was used to compare significance.

We further separated all patients into early-phase (recurrence recorded within 2 years of resection, n = 64) and late-phase groups (without recurrence recorded within 2 years of resection and patients with no recurrence, n = 25). We performed Kaplan–Meier estimate on these 2 groups comparing high or low infiltration of TILs and iMVD. The infiltration of CD3+ (*P* = .586) (Fig. 8A), CD8+ (*P* = .178) (Fig. 8B), FoxP3+ cells (*P* = .152) (Fig. 8C), and iMVD (*P* = .509) (Fig. 8D) had no significant influence on early-phase disease-free survival. The high infiltration of CD3+ (*P* = .014) (Fig. 9A) and CD8+ cells (*P* = .035) (Fig. 9B) significantly predicted better late-phase disease-free survival, but infiltration of FoxP3+ cells (*P* = .978) (Fig. 9C) and iMVD (*P* = .550) (Fig. 9D) could not significantly predict late-phase disease-free survival.

Univariate analyses showed no significant demographic variables for predicting early-phase disease-free survival, except for a tendency from white blood cell count (*P* = .072). (Table 5) For late-phase disease-free survival, tumor size (*P* = .076) and albumin (*P* = .056) showed potential predictabilities and were included in multivariate analysis with CD3+ (*P* = .014) and CD8+ (*P* = .035) cells. (Table 5) After the multivariate process, CD3+ (*P* = .012), tumor size (*P* = .032) and albumin (*P* = .007) were independent predictors for late-phase disease-free survival. (Table 6)

**Table 5:** Univariate Kaplan–Meier analysis on early- and late-phase DFS.

Variables	Early-phase DFS (*P* values)	Late-phase DFS (*P* values)
Gender	.436	.986
Age	.287	.963
HBV	.588	.258
Cirrhosis	.267	.107
AFP	.196	.837
Tumor size	.672	.076
Tumor multiplicity	.340	.760
Microvascular invasion	.243	n.a.
Macrovascular invasion	.329	n.a.
BCLC stage	.649	.285
Beyond milan criteria	.776	.255
albumin	.493	.056
ALT	.299	.187
AST	.832	.245
Bilirubin	.612	.129
WBC	.072	.407
HGB	.336	.166
PLT	.688	.526
CD3+ cells	.586	.014
CD8+ cells	.178	.035
FoxP3+ cells	.152	.978
iMVD	.509	.550

**Table 6:** Multivariate Cox regression of included variables on late-phase DFS.

Variables	Hazard ratio	95% Confidence interval	*P* values
CD3	0.036	0.003–0.481	.012
Tumor size	12.658	1.247–125.00	.032
Albumin	0.018	0.001–0.336	.007

## 4 Discussion

In this study, we found a correlation between iMVD and infiltration of CD3+ and CD8+ cells. We also confirmed the significant predictability of infiltration of CD3+ cells on the overall and late-phase disease-free survival after resection of HCC.

Many previous studies have suggested that the high intratumoral infiltration of CD3+ and CD8+ cells could better predict overall or disease-free survival,^[17–20]^ In the present study, we confirmed the influence of CD3+ and CD8+ cell infiltration on overall survival, but failed to prove their influence on disease-free survival. However, we noticed high recurrence rate within 2 years of resection in the current cohort. Since 2 years has been widely considered as a cut-off point for distinct mechanisms (metastasis or de novo) of intrahepatic recurrence after resection of HCC,^[21]^ we separated patients into early and late-phase disease-free survival groups, and found that high infiltration of CD3+ and CD8+ cells correlated with better late-phase disease-free survival, but not early-phase. A possible explanation for this could be that a major antitumor effect of the infiltrating CD3+ and CD8+ cells is preventing carcinogenesis rather than clearing existing cancer cells. Due to the high interaction effects between CD3+ and CD8+ cells, after multivariate analyses, CD8+ cells was excluded and CD3+ cells remained as independent predictors for overall and late-phase disease-free survival. Meanwhile, in our corhort, we also validated the independent predictabilities for disease-free survival from the well-known clinical variables for tumor aggressiveness including AFP, microvascular invasion, and tumor size, and those variables for impaired liver function including hepatitis B, ALT, and albumin.

In our results, we failed to obtain a significant predictability for the infiltration of FoxP3+ cells on disease-free or overall survival. Previous publications have indicated that high infiltration of FoxP3+ cells could predict worse overall and disease-free survival.^[22–26]^ In contrast, there are several studies that failed to validate the influence of FoxP3+ cells on survival.^[27,28]^ In this study, the number of FoxP3 expressed cells was notably lower than that of CD8, and was considerably correlated with CD8. Therefore, we infer that in our study most cases were predominantly CD8+ infiltrated, and the pro-tumor effect from FoxP3+ cells is mainly due to the suppression of immune effector cells. Hence, it could not be observed statistically in our study.

In our previous study on a western HCC cohort, we noticed that intratumoral CD3+, CD8+, CD20+, and CD66b+ cells were present predominantly around the intratumoral microvessels.^[14]^ We used perivascular regions to capture the hotspots for further survival analysis and validated the prognostic significance.^[14]^ However, it was too weak to prove a relationship between immune cell infiltration and neovascularization, which we were unable to quantify. In this study, we quantified neovascularization by measuring iMVD using the well-known Weidner's method. We proved that iMVD significantly correlated with TILs in clinical HCC tissue. This correlation is similar to that in other human solid tumors.^[8,29,30]^ Therefore, we suggest that neovascularization in HCC could be protective by promoting immune effector cell infiltration, which is dependent on the network of blood vessels. We further assume that for patients who possibly had high immune effector cell infiltration, the outcome of angiogenesis-targeted therapies, such as transarterial chemoembolization, sorafenib, regorafenib, and lenvatinib, may not be satisfactory.

Previous animal studies have revealed that Tregs could contribute to neovascularization through several mechanisms, including suppression of effector T cells and induction of chemokine CCL28 expression.^[9,31]^ In a previous clinical study, the author also observed a positive correlation between tumor-infiltrating Tregs and microvessel density in HCC.^[32]^ However, in our clinical HCC cohort, we could not validate the association between FoxP3+ cells and iMVD. The correlation between Tregs and microvessel density might have resulted from the probable interaction between them, where microvessels bring more T cells comprising Tregs into the tumor, and Tregs in turn promotes angiogenesis by the mechanisms mentioned above. In our study, we have provided evidence for the following: (a) iMVD correlated with high CD3+ cells, (b) CD3+ cells showed correlation with FoxP3+ cells, and (c) the direct significance of FoxP3+ cells and iMVD was not observed when the major subtype of T cells, CD8+ cells, were excluded.

The limitations of the current study were the relatively small sample size and one-point sampling without considering intratumoral heterogeneity, which might have resulted in the lack of statistical robustness.

## 5 Conclusion

In conclusion, our data suggests that increased iMVD could enrich more tumor-infiltrating CD3+ cells. Infiltrated CD3+ cells could help to better predict both the overall and late-phase disease-free survival after resection of HCC.

## Author contributions

**Acquisition of data:** Yuan Yang, Haiqing Wang.

**Analysis and interpretation of data:** Yuan Yang, Jingcheng Hao.

**Conceptualization and methodology:** Yuan Yang, Ning Fu, Jingcheng Hao.

**Development of methodology:** Yuan Yan, Jingcheng Hao.

**Funding Acquisition:** Yuan Yang, Jingcheng Hao.

**Writing, review and/or revision of the manuscript:** Yuan Yang, Ning Fu, Haiqing Wang, Jingcheng Hao.

## Supplementary Material

Supplemental Digital Content
