# Supplementary material for: Leukocytes infiltration correlates intratumoral microvessel density and influence overall and late-phase disease-free survival in hepatocellular carcinoma
Source: Medicine (Baltimore). 2021 Dec 3;100(48):e28135. doi: 10.1097/MD.0000000000028135 (PMC9191282; doi:10.1097/MD.0000000000028135)
Supplement: Supplemental Digital Content [file medi-100-e28135-s001.doc]

Supplementary Table 1.

|  | CD3 | | | CD8 | | | FoxP3 | | | CD31 | | |
| --- | --- | --- | --- | --- | --- | --- | --- | --- | --- | --- | --- | --- |
| Variables | High | Low | P | High | Low | P | High | Low | P | High | Low | P |
| Gender  (Male/Female) | 38/6 | 39/6 | 1.000 | 39/5 | 38/7 | 0.758 | 40/4 | 37/8 | 0.353 | 40/5 | 37/7 | 0.550 |
| Age (Years)  (Median (IQR)) | 51.50 (21.00) | 52.00 (16.50) | 0.166 | 54.00 (19.75) | 51.00 (17.50) | 0.136 | 50.00 (20.50) | 54.00 (17.00) | 0.571 | 52.00 (17.50) | 51.50 (20.75) | 0.452 |
| Hepatitis B | 33/11 | 38/7 | 0.302 | 33/11 | 38/7 | 0.302 | 37/7 | 34/11 | 0.430 | 37/8 | 34/10 | 0.606 |
| Cirrhosis | 33/11 | 30/15 | 0.486 | 31/13 | 32/13 | 1.000 | 31/13 | 32/13 | 1.000 | 30/15 | 33/11 | 0.486 |
| AFP | 50.50 (592.50) | 80.00 (5831.50) | 0.522 | 67.00 (797.75) | 67.00 (1417.50) | 0.997 | 298.00 (1860.25) | 19.60 (201.00) | **0.005** | 47.00 (3251.50) | 73.50 (701.46) | 0.961 |
| Tumor Size | 4.00 (3.00) | 6.00 (6.25) | **0.026** | 4.00 (3.00) | 6.00 (5.25) | 0.110 | 5.00 (3.75) | 5.00 (5.00) | 0.720 | 5.00 (5.00) | 5.50 (5.00) | 0.964 |
| Tumor Multiplicity | 4/44 | 8/37 | 0.353 | 5/39 | 7/38 | 0.758 | 5/39 | 7/38 | 0.758 | 5/40 | 7/37 | 0.550 |
| Microvascular Invasion | 1/43 | 4/41 | 0.361 | 1/43 | 5/40 | 0.203 | 2/42 | 3/42 | 1.000 | 1/44 | 4/40 | 0.203 |
| Macrovascular Invasion | 0/44 | 6/39 | **0.026** | 2/42 | 3/42 | 1.000 | 3/41 | 3/42 | 1.000 | 0/45 | 6/38 | **0.012** |
| BCLC Stage () | 3/32/4/5 | 2/18/11/14 | **0.009** | 3/27/7/7 | 2/23/8/12 | 0.595 | 1/26/7/10 | 4/24/8/9 | 0.575 | 1/29/9/6 | 4/21/6/13 | 0.100 |
| Beyond Milan Criteria | 22/22 | 31/14 | 0.086 | 22/22 | 31/14 | 0.086 | 26/18 | 27/18 | 1.000 | 25/20 | 28/16 | 0.519 |
| Albumin | 41.35 (6.05) | 41.00 (5.60) | 0.977 | 42.30 (6.35) | 40.90  (5.45) | 0.485 | 40.25 (6.23) | 42.20 (4.35) | 0.135 | 40.50 (5.90) | 41.20 (5.40) | 0.597 |
| ALT | 34.50 (36.00) | 41.00 (44.00) | 0.554 | 35.50 (38.75) | 38.00 (41.50) | 0.818 | 37.00 (43.75) | 37.00 (35.50) | 0.358 | 37.00 (40.50) | 36.00 (34.50) | 0.749 |
| AST | 34.00 (20.25) | 43.00 (43.00) | 0.101 | 34.00 (22.50) | 41.00 (38.00) | 0.178 | 38.00 (31.75) | 35.00 (23.50) | 0.588 | 35.00 (24.00) | 42.00 (34.25) | 0.141 |
| Bilirubin | 16.65 (11.63) | 17.10 (6.80) | 0.688 | 15.95 (9.60) | 17.50 (7.75) | 0.132 | 17.45 (11.40) | 16.10 (7.55) | 0.417 | 17.00 (8.45) | 17.05 (8.55) | 0.549 |
| WBC | 5.38 (2.45) | 5.30 (3.17) | 0.886 | 5.37 (2.95) | 5.30 (3.10) | 0.367 | 5.37 (2.93) | 5.30 (2.63) | 0.977 | 5.39 (2.89) | 5.35 (3.04) | 0.746 |
| HGB | 136.00 (25.50) | 140.00 (24.50) | 0.448 | 136.50 (24.00) | 139.00 (25.50) | 0.371 | 137.50 (22.25) | 140.00 (27.00) | 0.414 | 139.00 (29.00) | 138.50 (23.75) | 0.805 |
| PLT | 112.00 (55.25) | 147.00 (114.50) | **0.040** | 112.00 (61.25) | 145.00 (100.00) | 0.097 | 115.00 (67.75) | 129.00 (111.5) | 0.694 | 124.00 (87.50) | 123.50 (90.00) | 0.533 |
|  |  |  |  |  |  |  |  |  |  |  |  |  |
